# Seroprevalence of SARS-CoV-2 in Patients with Multiple Sclerosis under Disease-Modifying Therapies: A Multi-Centre Study

**DOI:** 10.3390/jcm12237243

**Published:** 2023-11-23

**Authors:** Agustín Sancho-Saldaña, Anna Gil-Sánchez, Cristina González-Mingot, Silvia Peralta, Maria Jose Solana, Pascual Torres, Alba Juanes, Laura Quibus, Emilio Ruiz, Eduardo Sanpedro, Bibiana Quirant-Sánchez, Eva Martínez-Cáceres, Cristina Ramo Tello, Silvia Presas-Rodríguez, Sebatián García Rubio, Beatriz Pardiñas Baron, Lluís Ramió-Torrentà, Javier Sotoca, Inés González-Suárez, Sara Eichau, José M. Prieto-González, Maria Rosario Blasco Quilez, Julia Sabín-Muñoz, Antonio José Sánchez-López, Gloria Llorens Calatayud, Carmen Calles, Ángel Pérez Sempere, Moises Garcés, Olga Carmona, Ester Moral, José Vicente Hervás, Yolanda Blanco, Nuria Sola-Valls, Nieves Tellez Lara, Lucía Forero, Luis Brieva

**Affiliations:** 1 Neurology Department, Hospital Universitario Arnau de Vilanova, Institut de Recerca Biomèdica de Lleida-IRBLleida, 25198 Lleida, Spain; agustinsanchosaldana@gmail.com (A.S.-S.); cgonzalezm.lleida.ics@gencat.cat (C.G.-M.); solanamoga@gmail.com (M.J.S.); lquibus.lleida.ics@gencat.cat (L.Q.); eruizf.lleida.ics@gencat.cat (E.R.); emurillo.lleida.ics@gencat.cat (E.S.); 2 Neuroimmunology Group, Department of Medicine, Institut de Recerca Biomèdica de Lleida-IRBLleida, 25198 Lleida, Spain; annagil.78@gmail.com (A.G.-S.); ptorres@irblleida.cat (P.T.); albajuanes@xij.gencat.cat (A.J.); 3 Multiple Sclerosis Foundation from Lleida, 25198 Lleida, Spain; speralta.lleida.ics@gencat.cat; 4 Metabolic Physiopathology Group, Department of Experimental Medicine, Institut de Recerca Biomèdica de Lleida-IRBLleida, 25198 Lleida, Spain; 5 Immunology Division, Hospital Germans Trias i Pujol, LCMN, 08916 Badalona, Spain; bquirant.germanstrias@gencat.cat (B.Q.-S.); emmartinez.germanstrias@gencat.cat (E.M.-C.); 6 Department of Cell Biology, Physiology, Immunology, Autonomous University, Bellaterra, 08193 Barcelona, Spain; 7 Multiple Sclerosis and Clinical Neuroimmunology Unit, Neurosciences Department, Hospital Germans Trias i Pujol, 08916 Badalona, Spain; cramot@gmail.com (C.R.T.); spresas@igtp.cat (S.P.-R.); 8 Neurology Department, Hospital Universitario Miguel Servet, 50009 Zaragoza, Spain; sebastiangarciarubio@gmail.com (S.G.R.); bpardinnas@salud.aragon.es (B.P.B.); 9 Girona Neuroimmunology and Multiple Sclerosis Unit, Neurology Department, Dr. Josep Trueta University Hospital, 17007 Girona, Spain; llramio@idibgi.org; 10 Neurodegeneration and Neuroinflammation Research Group, IDIBGI, 17190 Salt, Spain; 11 Medical Sciences Department, University of Girona, 17071 Girona, Spain; 12 Neurology Department, Hospital Universitari MutuaTerrassa, 08035 Barcelona, Spain; jasotoc@gmail.com; 13 Department of Neurology, Complejo Hospitalario Universitario de Vigo, Calle Clara Campoamor, 341, 36213 Vigo, Spain; igonsua@gmail.com; 14 Multiple Sclerosis Unit, Hospital Universitario Virgen Macarena, 41009 Seville, Spain; saraeichau@gmail.com; 15 Fundación Instituto de Investigación Sanitaria de Santiago (IDIS), 15706 Santiago de Compostela, Spain; josemaoscar.prieto@usc.es; 16 Neuroimmunology Unit, Clínica Puerta de Hierro, Universidad Autónoma de Madrid, 28222 Madrid, Spain; mariarosario.blasco@salud.madrid.org (M.R.B.Q.); julia.sabin.m@gmail.com (J.S.-M.); 17 Biobank, Puerta de Hierro-Segovia de Arana Health Research Institute, 28222 Madrid, Spain; 18 Neuroimmunology Unit, Puerta de Hierro-Segovia de Arana Health Research Institute, 28222 Madrid, Spain; 19 Department of Neurology, Hospital Mateu Orfila, 07703 Mahón, Spain; glori_llorens@hotmail.com; 20 Department of Neurology, Hospital Universitari Son Espases, 07120 Palma de Mallorca, Spain; mcalles22@yahoo.es; 21 Department of Neurology, Hospital General Universitario Dr. Balmis de Alicante, Universidad Miguel Hernández de Elche, 03202 Elche, Spain; aperezs@mac.com; 22 Department of Neurology, Hospital Clínico Universitario Lozano Blesa, 50009 Zaragoza, Spain; moisesgarce@gmail.com; 23 Department of Neurology, Fundació Salut Empordà, 17600 Girona, Spain; occodina@gmail.com; 24 Hospital de Sant Joan Despí Moisès Broggi, 08970 Sant Joan Despí, Spain; estermoral@yahoo.es (E.M.); josevicente.hervas.garcia@gmail.com (J.V.H.); 25 Neuroimmunology and Multiple Sclerosis Unit, Neurology Service, Hospital Clinic de Barcelona, 08036 Barcelona, Spain; yblanco@clinic.cat; 26 Department of Neurology, Hospital Universitari Sant Joan de Reus, 43204 Tarragona, Spain; nuria.sola@gmail.com; 27 Department of Neurology, Hospital Clínico Universitario de Valladolid, 47003 Valladolid, Spain; tellezlara@gmail.com; 28 Department of Neurology, Hospital Puerta del Mar, 11009 Cádiz, Spain; lucia.forero.diaz@hotmail.es

**Keywords:** Multiple Sclerosis, COVID-19, SARS-CoV-2, DMT, seroprevalence

## Abstract

Background: The EMCOVID project conducted a multi-centre cohort study to investigate the impact of COVID-19 on patients with Multiple Sclerosis (pwMS) receiving disease-modifying therapies (DMTs). The study aimed to evaluate the seroprevalence and persistence of SARS-CoV-2 antibodies in MS patients enrolled in the EMCOVID database. The DMTs were used to manage MS by reducing relapses, lesion accumulation, and disability progression. However, concerns arose regarding the susceptibility of pwMS to COVID-19 due to potential interactions between SARS-CoV-2 and the immune system, as well as the immunomodulatory effects of DMTs. Methods: This prospective observational study utilized data from a Multiple Sclerosis and COVID-19 (EMCOVID-19) study. Demographic characteristics, MS history, laboratory data, SARS-CoV-2 serology, and symptoms of COVID-19 were extracted for pwMS receiving any type of DMT. The relationship between demographics, MS phenotype, DMTs, and COVID-19 was evaluated. The evolution of SARS-CoV-2 antibodies over a 6-month period was also assessed. Results: The study included 709 pwMS, with 376 patients providing samples at the 6-month follow-up visit. The seroprevalence of SARS-CoV-2 antibodies was higher among pwMS than the general population, with Interferon treatment being significantly associated with greater seroprevalence (16.9% vs. 8.4%; *p* 0.003). However, no other specific DMT showed a significant association with antibody presence. A total of 32 patients (8.5%) tested positive for IgG, IgM, or IgA antibodies against SARS-CoV-2 at baseline, but then tested negative at 6 months. Most of the pwMS in the cohort were asymptomatic for COVID-19 and, even among symptomatic cases, the prognosis was generally favourable. Conclusion: pwMS undergoing DMTs exhibited a higher seroprevalence of COVID-19 than the general population. Interferon treatment was associated with a higher seroprevalence, suggesting a more robust humoral response. This study provides valuable insights into the seroprevalence and persistence of SARS-CoV-2 antibodies in pwMS and contributes to our understanding of the impact of COVID-19 amongst this population.

## 1. Introduction

The EMCOVID project is a Spanish multi-centre cohort study that prospectively collected data on patients with Multiple Sclerosis (PwMS) under disease-modifying therapies (DMTs) and the COVID-19 pandemic, focusing on a serological test for SARS-CoV-2.

DMTs are used to treat PwMS in order to reduce the frequency and severity of relapses and the accumulation of lesions detected with magnetic resonance imaging, and to slow disability progression [1].

However, there was growing concern about how the COVID-19 pandemic could impact MS due to the possibility of increased susceptibility to COVID-19, given the existence of numerous potential interactions between SARS-CoV-2 and the immune system, the immunomodulatory/immunosuppressive effects of DMTs, and—in a posterior phase—the development of adequate humoral and cellular immunity after SARS-CoV-2 exposure [2].

Growing evidence supports the association between SARS-CoV-2 viral infection and the risk of demyelination in both the peripheral and central nervous systems [3]. This demyelination is caused by several proposed mechanisms [4]:

(1) Inflammation and Autoimmune Response: Virtually all components of the inflammatory cascade observed in MS have been identified in COVID-19 patients. It has been discovered that cytotoxic T lymphocytes and pro-inflammatory cytokines can cross the blood–brain barrier, influencing innate immune cells within the central nervous system, including macrophages, microglia, and astrocytes, thereby prompting their pro-inflammatory activation and initiating immune-mediated demyelination.
(2) Direct Effect of the Virus on Oligodendrocytes: The impact of the SARS-CoV-2 virus on oligodendrocyte functionality and survival remains unexplored, but several factors strongly suggest its potential influence. Notably, SARS-CoV-2 has been observed to breach the blood–brain barrier, and an experimental study has demonstrated that infected oligodendrocytes, while surviving, exhibit altered gene expression near demyelinated regions, contributing to chronic inflammation [5].
(3) Cerebral Blood Flow Impairment: Changes in brain microstructure, cerebral blood flow, and tract parameters have shown significant correlations with inflammatory markers such as C-reactive protein, procalcitonin, and interleukin-6 [6]. Multiple studies suggest that the SARS-CoV-2 virus can lead to myelin damage, oligodendrocyte death, and disruptions in neurological function due to impaired respiration, resulting in hypoxia, cerebral ischemia, and an inflammatory response to viral infection [4].

Although our understanding of the significance of antibody response to SARS-CoV-2 is still—at present—limited, serological tests have proved useful tools for diagnosing prior SARS-CoV-2 infection and for potentially reflecting acquired protection [7].

This protection may be reduced in PwMS receiving DMTs and be reflected by a weaker antibody response to SARS-CoV-2 [8]. However, it has also been suggested that some DMTs could be safer than others, and even more protective, according to their mechanism of action [9,10].

Some preliminary data suggest that PwMS do not have an increased risk of COVID-19 infection compared to the general population. However, it seems that the risk and severity of COVID-19 infection in PwMS may increase in those taking anti-CD20 therapies [10,11,12].

To date, very limited data are available regarding either the rate of seroconversion in large cohorts of PwMS previously infected with SARS-CoV-2 or its persistence (over time).

Spain was one of the European countries most affected by the COVID-19 pandemic. Serological surveys were a valuable tool to assess the extent of the epidemic, given the existence of asymptomatic cases and little access to diagnostic tests. ENE-COVID was a nationwide population-based study aiming to estimate the seroprevalence of SARS-CoV-2 infection in Spain at the national and regional level [13].

The EM-COVID project is a Spanish multi-centre cohort study that prospectively collected data on patients with Multiple Sclerosis (pwMS) under disease-modifying therapies (DMTs) and the COVID-19 pandemic, focusing on a serological test for SARS-Cov-2.

This study aims to evaluate the seroprevalence of anti-SARS-CoV-2 antibodies as well as its persistence over time, based on DMTs, in a sample of PwMS who were enrolled and then followed longitudinally via the EMCOVID database. We also compare these data with the seroprevalence of the general population obtained via the ENE-COVID.

## 2. Material and Methods

### 2.1. Study Design and Patients

This was a multi-centre prospective observational study based on EMCOVID-19 (Esclerosis Múltiple y COVID-19, in Spanish). It forms part of an ongoing, prospective study, conducted at 20 centres in Spain from April to September 2020, whose aim is to evaluate the seroprevalence of SARS-CoV-2 over time, in a large cohort of pwMS treated with DMT prior to SARS-CoV-2 vaccination.

The EMCOVID-19 study encompassed two visits (baseline and at 6 months) in which the latest, or most recent, clinical manifestations of COVID-19 and MS were assessed and a blood sample was taken to determine the presence of SARS-CoV-2 antibodies.

All patients diagnosed with MS and treated with any type of DMT were included in the EMCOVID database. Data were extracted from the baseline and 6-month visits of the EMCOVID-19 study. Basal characteristics (sex, age, pregnancy, smoker history), MS history data (MS phenotype, Expanded Disability Status Scale (EDSS), time from MS diagnosis, time from first symptoms, time from the last relapse, use of glucocorticoids in the last 3 months, current DMT), laboratory data (lymphocyte count), and symptoms of COVID-19 were recorded. The relationship between any of these characteristics and the presence of SARS-CoV-2 antibodies in serum was then analysed. Lymphopenia was considered when the absolute lymphocyte count was <1000/μL.

Patients with IgG, IgM, or IgA antibodies against SARS-CoV-2 were considered to constitute confirmed cases of SARS-CoV-2 infection and they were classified as symptomatic or asymptomatic based on the presence or absence of compatible symptoms in the recent past.

The serological status between the two time points was codified as “No change” (Seropositivity basal-Seropositivity 6 months, or Seronegativity basal-Seronegativity 6 months), Seropositivity to Seronegativity, or Seronegativity to Seropositivity

A nationwide, population-based, seroepidemiological study provided epidemiological data of COVID-19 cases based on a serological analysis (Spanish Ministry of Health, Consumer Affairs and Social Welfare and ENE-COVID) [13].

A total of 35,883 households were selected through a two-stage random sampling method, stratified by province and municipality size. All residents were invited to participate in the study. Between 27 April and 11 May 2020, a cohort of 61,075 participants, constituting 75.1% of the contacted individuals within the selected households, completed a questionnaire assessing their history of COVID-19-compatible symptoms and associated risk factors. Subsequently, they underwent point-of-care antibody testing (based on igG) and, upon consent, provided blood samples for further examination using a chemiluminescent microparticle immunoassay, enabling the qualitative detection of IgG against SARS-CoV-2 nucleoprotein.

**Figure 1:**
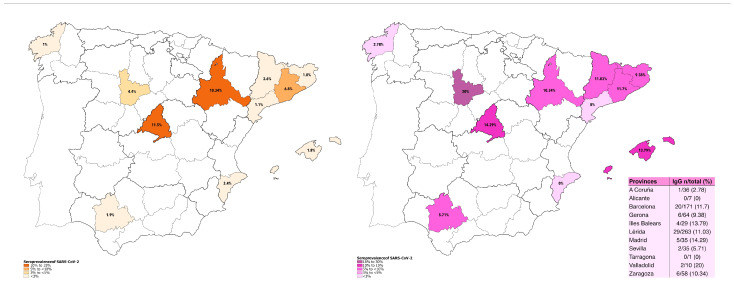
igG antibodies by province in the general population (**left**) and pwMS (**right**).

The results of seroprevalence obtained from our study (EM-COVID) were compared with those obtained for the general population (ENE-COVID) (Figure 1).

### 2.2. Blood Samples

Blood samples were extracted with a peripheral puncture from March 2020 to September 2020: before COVID-19 vaccination began in Spain (28 December 2020). The samples obtained were centrifuged and then frozen at −80 °C.

SARS-CoV-2 antibodies (IgG, IgM, and IgA anti-SARS-CoV-2) were analysed using an enzyme-linked immuno-sorbent assay (ELISA) containing a pool of S and N recombinant antigens (Diapro^®^, Sesto San Giovanni, Italy), according to the manufacturer’s instructions. In addition, those sera-positive for IgG anti-SARS-CoV-2 antibodies were re-analysed with an independent confirmatory ELISA assay that separately measured IgG antibodies to spike glycoprotein-1 (S1), spike glycoprotein-2 (S2), or N antigens.

Results were expressed with an index value, calculated as the ratio between the optical density (OD) of each sample and the OD of the cut-off reagent provided by the manufacturer. Index values ≥1.1 were considered positive and <1.1 were classified as negative.

### 2.3. Statistical Analysis

A descriptive analysis was performed using absolute frequencies (percentage) for categorical variables, while numeric ones were described through the median [interquartile range—IQR].

Demographic and clinical differences between seropositive and seronegative patients were assessed with a bivariate analysis: for quantitative variables with normal distribution and homoscedasticity between groups, a t-test was performed (parametric); otherwise, the Mann–Whitney U test (non-parametric) was used. The Anderson–Darling test and Fligner–Killen test were used to asses normality and homoscedasticity, respectively. Associations between patient groups and categorical variables were assessed through the chi-square test or Fisher exact test (when expected frequencies of more than 20% of cells were lower than 5).

For comparing the serological test results between the two time points (basal and 6 months), the McNemar test for paired proportions was applied. For evaluating the effect of therapy in changes of serologic status between the two time points, the chi-square or Fisher exact test was applied.

All statistical analyses were performed using R software v4.2.1. Statistical tests applied were two-tailed and the significance level threshold was set at 0.05.

### 2.4. Ethics

This study was subject to thorough evaluation and approval by the Ethics Committee, as detailed below:

Ethics Committee Name: Clinical Research Ethics Committee of Arnau de Vilanova University Hospital in Lleida.

Approval Code: CEIC-2253.

Approval Date: 04/16/2020.

## 3. Results

A total of 709 patients were included in the initial data, from which we obtained samples relating to 376 at 6 months. The median age was 43 [36–50] years old and 481 (68.1%) of the patients were female. Related to the clinical phenotype of MS, 600 patients (84.7%) had relapsing–remitting Multiple Sclerosis (RRMS), 67 (9.5%) had secondary progressive Multiple Sclerosis (SPMS), and 41 (5.8%) had primary progressive Multiple Sclerosis (PPMS). In total, 598 patients (86.7%) had not exhibited any signs of relapse in the previous year, and only 14 (2%) received glucocorticoids to treat a relapse in the previous 3 months.

In total, 268 patients (37.8%) were taking some of the first-line disease-modifying treatments (DMTs), Interferon, Copaxone, Teriflunomide, or Dimethyl fumarate, and 441 (62.2%) were taking second-line DMTs, Cladribine, Fingolimod, Alemtuzumab, Natalizumab, Ocrelizumab, or Rituximab. More information about the baseline characteristics and treatments is presented in Table 1.

In total, 157 patients (22.1%) had lymphopenia (<1000 lymphocytes), of whom 23 (14.6%) had severe lymphopenia (grade 4; <200 lymphocytes).

At the baseline, 136 (19.2%) patients had positive IgG, IgM, or IgA antibodies against SARS-CoV-2; 78/376 (20.7%) had them at 6 months, while 165/431 (38.3%) had them at any time (baseline or 6 months). Data related to antibodies against SARS-CoV-2 are shown in Table 2. Six patients at baseline and three at 6 months had positive PCRs, but they did not have antibodies against SARS-CoV-2. In total, 4 of them were on anti-CD20 DMTs (2 on Ocrelizumab and 2 on Rituximab), 2 were on Copaxone, 1 on Natalizumab, 1 on Fingolimod, and 1 on Teriflunomide.

When comparing the serological test results at both time points (baseline and 6 months), no significant differences in the proportions of serological outcomes were observed (*p* = 0.798). A total of 49 patients (13%) were seropositive throughout the follow-up period; however, 32 (8.5%) tested positive for IgG, IgM, or IgA antibodies against SARS-CoV-2 at the baseline but were negative at 6 months. Additionally, our analysis did not reveal any discernible association between treatment and serological status at the two time points, as summarized in Table 3.

At baseline, 78 (11%) patients had had some symptoms related to COVID-19 but only 1 of these (1.2%) had required hospitalization. This patient was being treated with Ocrelizumab and had presented with fever, moderate dyspnoea, and bilateral pneumonia. He received Hydroxychloroquine and oxygen therapy, and made a good recovery after 15 days of hospitalization. At 6 months, 69 patients (11%) had symptoms related to COVID-19, with these being mild in 67 (97.1%) cases.

The binary analysis showing differences between seropositive and seronegative patients is presented in Table 4. Patients taking Interferon were significantly associated with the presence of SARS-CoV-2 antibodies (16.9% vs. 8.4%; *p* 0.003). We did not find any statistical association with the rest of the DMTs or with any other characteristics.

In the ENE-COVID study conducted between 27 April and 11 May 2020, the seroprevalence rates for the entire nation were reported as 5.0% via the point-of-care test and 4.6% through the immunoassay. These estimations exhibited substantial regional variation, with notably higher rates detected in seven provinces located in the central part of Spain, including Madrid, where seroprevalence exceeded 10% for both the point-of-care test and immunoassay individually. Along the coastal provinces, seroprevalence surpassed 5% solely in Barcelona (Figure 1). The seroprevalence estimates derived from both testing methods were consistently similar.

## 4. Discussion

Considerable literature has been dedicated to elucidating the immune response to various anti-SARS-CoV-2 vaccines in the general population, with particular focus on pwMS and the potential detrimental effects of DMTs employed and relating to SARS-CoV-2 protection [14]. However, very few studies have so far examined immune responses in pWMS during the COVID-19 pandemic prior to the administration of vaccines.

Historical records indicate that prior pandemics were caused by other coronaviruses [15]. The potential resurgence of this, or other related viruses, and their ability to trigger another pandemic remain unknown. Comprehensive serological data preceding SARS-CoV-2 vaccination would be invaluable for informed decision making relating to the treatment of both the general population and individuals with autoimmune disorders and/or subject to immunosuppressive regimens.

This study evaluated the seroprevalence of SARS-CoV-2 antibodies in PwMS taking DMTs, regardless of whether or not they had COVID-19 symptoms. This was conducted according to a pre-planned schedule.

The serological response to SARS-CoV-2 infection and vaccination is still being investigated. To date, most COVID-19 serological studies have focused on symptomatic cases and there has been conflicting data regarding the host humoral response to the virus, especially in asymptomatic and mild cases [16]. It should be added that most studies have been conducted in healthy people without autoimmune diseases. Furthermore, humoral immune responses in PwMS may vary and be conditioned with each specific DMT, depending on its mechanism of action.

It is worth highlighting that the pwMS receiving DMT exhibited a higher seroprevalence when compared to the general population without MS. At the study’s baseline, 19.2% of pwMS tested positive for IgG, IgM, or IgA antibodies against SARS-CoV-2 (10.6% tested positive just to IgG). In contrast, during the same timeframe, the general Spanish population registered a seropositivity rate for SARS-CoV-2 of 5.0% (point-of-care test), 4.6% (immunoassay), or 6.2% (either test positive) [13]. Furthermore, a province-based comparison reveals a heightened seroprevalence in pwMS when compared to the general population (Figure 1).

Based on the available data, it appears that pwMS receiving DMTs may exhibit increased susceptibility to SARS-CoV-2 infection. Notwithstanding this potential vulnerability, pwMS did not manifest any worse outcomes than other patients and, in fact, in a significant number of cases, they had detectable antibodies, even in the absence of prior history of symptomatic COVID-19 infection.

Population-based studies on the incidence of COVID-19 in pwMS compared to in the general population remain relatively scarce. Even so, a Scottish study [17] reported similar rates of COVID-19 infection in both populations and a Brazilian study, involving 11,560 pwMS, found similar results [18]. These studies used PCR tests to define COVID-19 cases instead of a serological test, which could perhaps explain the disparity with our results. In contrast, a survey study performed in Barcelona found an almost two-fold increase in the incidence of COVID-19 in pwMS with respect to the general population [19].

Although not statistically significant, female sex tended to be associated with increased seropositivity (*p* = 0.056). Along these lines, a case–control study analysing the risk of COVID-19 in pwMS reported a higher degree of susceptibility in younger age groups and associated with patients being female, having more comorbidities, receiving natalizumab, and/or receiving an escalating treatment strategy [20]. However, the ENE-COVID study showed no discernible differences observed with gender.

As previously noted, in our study, neither previous lymphocyte counts nor the degree of lymphopenia was associated with a greater risk of COVID-19 [21].

Regarding DMTs, PwMS treated with Interferon were significantly associated with a higher rate of seropositivity; this confirmed our previous findings from a single centre report [22]. This could be explained with Interferon having less effect on the humoral immune system, resulting in a more appropriate serological response rather than an increased risk of COVID-19 infection.

Similarly, a Polish study showed that PwMS treated with either dimethyl fumarate, Interferon, or glatiramer acetate efficiently produced antibodies against SARS-CoV-2 both after infection and vaccination [23]. Interferon was also shown to confer protection against severe COVID-19 infection in a case–control study [20].

B-cell depletion therapies, such us Ocrelizumab and Rituximab, may result in a reduced probability of patients generating a detectable neutralizing antibody titre. As a result, anti-CD20 treatments have been some of the therapies that have provoked most concern in the COVID-19 era [24].

However, in our study, we did not find a lower level of COVID-19 seroprevalence in pwMs taking anti-CD20 therapies. In fact, evidence relating to the impact of anti-CD20 therapies in the COVID-19 era conflicts with some studies that have shown lower frequencies of positive serological tests in anti-CD20-treated pwMS [4,17,20,25]. The COVID-19 prognosis in these patients has also been conflictive. Several studies suggest that anti-CD20 therapy may be a risk factor for severe COVID-19. A multi-centre study involving 28 countries found consistent associations between anti-CD20 treatments and the risk of requiring hospital and intensive care in pwMS with COVID-19 infection [26]. An Italian study found that a therapy involving an anti-CD20 agent was associated with an increased risk of severe COVID-19 in 844 pwMS [11]. Other studies, however, found no association between exposure to anti-CD20 agents and the severity of COVID-19 infection [10,27]. Robust memory T-cell responses in antibody-seronegative cases could explain how immunity is achieved in this type of patient [25,28]. It would also suggest that serological responses to anti-SARS-CoV-2 vaccines have not been very effective, even though cellular responses may have been preserved [29].

We did not find any significant association between the other DMTs and antibodies to SARS-CoV-2 either. The application of immunosuppressive drugs did not increase the risk of infection or its severity compared with immunomodulatory drugs.

In our cohort, 13% of patients were consistently seropositive throughout the follow-up period (both at baseline and at 6 months), while 8.5% tested positive for IgG, IgM, or IgA antibodies against SARS-CoV-2 at baseline, but negative at 6 months. We did not find statistically significant associations with any specific DMT, although the proportion of patients with each specific DMT associated with serological information at 6 months was low, implying that this finding should be interpretated with a degree of caution.

Most of the PwMS in our cohort (89%) were asymptomatic for COVID-19. Of those who were symptomatic, only three had a severe course and were subsequently found to have antibodies. We did not find any correlation between having had compatible symptoms or a PCR test for COVID-19 and developing antibodies against SARS-CoV-2.

Based on the hypothesis that an overactive immune response could cause a clinical deterioration in COVID-19 infection, it has been suggested that some DMTs could protect against certain COVID-19-associated complications [30,31].

Previous studies that evaluated the course of COVID-19 in pwMS treated with DMTs indicated a relatively mild course of infection in most cases [10,32]. General risk factors, such as old age, obesity, and disability, have also been associated with worse courses of COVID-19 in pwMS [10,20,33]. In our study, however, none of these comorbidities were associated with a higher susceptibility to COVID-19 infection or to worse prognoses.

This study has its strengths and limitations. The first strength derives from it being a multi-centre study with quite a large sample size and from its prospective observational nature. Furthermore, the ELISA test used in the analysis is more sensitive to antibodies against SARS-CoV-2 than the techniques used in many other studies. This allowed us to detect the majority of COVID-19 exposure, regardless of the time of infection or its severity. Another strength of this study was the availability of a reference population for the same time period and epidemiological context as the ENECOVID study.

Our study had several limitations. Although we used data from the ENECOVID study as our reference population, we did not have a true control group with which to compare our findings. In addition, we did not use a quantitative analysis to measure antibody levels; this could have provided more information in some cases (especially regarding the relationship between antibody titres and their persistence at 6 months). Finally, we did not have the serologies of all the patients at 6 months, with the subsequent loss of relevant information.

In conclusion, according to our data, pwMs exhibited a higher seroprevalence of COVID-19 than general populations in other serological studies. Despite this, prognoses were generally good, with a high proportion of asymptomatic patients. Immunosuppression deriving from some DMTs could make pwMS more susceptible to COVID-19 infection, but would not result in less efficient immunological responses to fight the virus. Interferon was the only DMT associated with greater seroprevalence, implying a competent humoral response to COVID-19. None of the DMTs were associated with either the persistence or disappearance of antibodies at 6 months.

## Figures and Tables

**Table 1:** Demographic, clinical characteristics and DMT.

	Basal (N = 709)
Sex	
Female	481/706 (68.1%)
Male	225/706 (31.9%)
Age (years)	
Median [IQR]	43 [36–50]
Weight	
Underweight	12/469 (2.5%)
Healthy weight	269/469 (57.3%)
Overweight	113/469 (24.1%)
Obesity	75/469 (16%)
Hypertension	
	59 (8.3%)
Diabetes	
	19 (2.7%)
Ethnicity	
Asian	2 (0.3%)
Black or African American	2 (0.3%)
Caucasian	682 (96.2%)
Other	23 (3.2%)
Pregnancy: Basal (if female)	
Yes	6 (0.8%)
Smoker history: Basal	
Current smoker	134/602 (22.3%)
Former smoker	125/602 (20.8%)
Never smoked	343/602 (57.0%)
Alcohol: Basal	
Never used alcohol	333 (47%)
Occasional consumption	362 (51.1%)
Regular consumption	14 (2%)
MS type: Basal	
Primary progressive MS (PPMS)	41/708 (5.8%)
Relapsing remitting MS (RRMS)	600/708 (84.7%)
Secondary progressive MS (SPMS)	67/708 (9.5%)
EDSS	
Median [IQR]	2 [1–4]
Relapses in previous year	
	92/690 (13.3%)
Steroids in last 3 months, n (%)	
	14 (2%)
First-line DMT, n (%)	
Interferon	71 (10%)
Copaxone	28 (3.9%)
Teriflunomide	55 (7.8%)
Dimethyl	114 (16.1%)
Second-line DMT, n (%)	
Cladribine	43 (6.1%)
Fingolimod	47 (6.6%)
Alemtuzumab	69 (9.7%)
Natalizumab	124 (17.5%)
Ocrelizumab	109 (14.5%)
Rituximab	55 (7.8%)
COVID-19 symptoms	
Asymptomatic	631 (89.0%)
Symptomatic	78 (11%)
Mild	77 (98.7%)
Severe	1 (1.3%)
Lymphopenia	
	157 (22.1%)
≤200 Grade 4	23 (3.2%)
201–500 Grade 3	22 (3.1%)
501–800 Grade 2	42 (5.9%)
801–1000 Grade 1	70 (9.9%)
No lymphopenia	552 (77.9%)

**Table 2:** PCR and distribution of antibodies against SARS-CoV-2.

	Basal (N = 709)	6 Months (N = 376)
PCR		
Negative	70/89 (78.6%)	35/59 (59.3%)
Positive	19/89 (21.3%)	24/59 (40.7%)
Antibodies—IgG		
Negative	634 (89.4%)	334 (88.8%)
Positive	75 (10.6%)	42 (11.2%)
Antibodies—IgM		
Negative	623 (87.9%)	322 (85.6%)
Positive	86 (12.1%)	54 (14.4%)
Antibodies—IgA		
Negative	661 (93.2%)	342 (91%)
Positive	48 (6.8%)	34 (9%)
Seropositive igA or igG or igM	136 (19.2%)	78 (20.7%)

**Table 3:** Evolution of serological status according to DMT.

Variable	Total (N = 376)	No Change (N = 315)	Positive to Negative (N = 32)	Negative to Positive (N = 29)	*p*
DMT					0.842
Copaxone	13 (3.46%)	13 (4.13%)	0 (0.00%)	0 (0.00%)	
Dimethyl	8 (27.59%)	46 (14.60%)	5 (15.62%)	59 (15.69%)	
Interferon	43 (11.44%)	35 (11.11%)	7 (21.88%)	1 (3.45%)	
Teriflunomide	36 (9.57%)	29 (9.21%)	3 (9.38%)	4 (13.79%)	
Alentuzumab	18 (4.79%)	16 (5.08%)	1 (3.12%)	1 (3.45%)	
Cladribina	27 (7.18%)	24 (7.62%)	1 (3.12%)	2 (6.90%)	
Fingolimod	30 (7.98%)	23 (7.30%)	4 (12.50%)	3 (10.34%)	
Natalizumab	63 (16.76%)	54 (17.14%)	5 (15.62%)	4 (13.79%)	
Ocrelizumab	64 (17.02%)	54 (17.14%)	5 (15.62%)	5 (17.24%)	
Rituximab	23 (6.12%)	21 (6.67%)	1 (3.12%)	1 (3.45%)	

DMT: Disease-Modifying Therapy.

**Table 4:** Demographic and clinical characteristics, DMT, and COVID-19 immune status.

	Seropositive (N = 136)	Seronegative (N = 573)	*p*
Age (years) (median, [RIQ])	45 [38–50]	43 [36–50]	0.25
Sex (N)			0.056
Female (481)	102 (21.2%)	379 (78.8%)	
Male (225)	34 (15.1%)	191 (84.9%)	
EDSS (median, [RIQ])	2 [1.0–4.0]	2 [0.0–3.5]	0.219
Current smoker (N = 134)	24 (17.9%)	110 (82.1%)	0.412
MS type (N)			0.938
PPMS (41)	7 (17.1%)	34 (82.9%)	
RRMS (600)	116 (19.3%)	484 (80.7%)	
SPMS (67)	13 (19.4%)	54 (80.6%)	
Hypertension (N = 59)	13 (22%)	46 (78%)	0.561
Diabetes (N = 19)	3 (15.8%)	16 (84.2%)	1.00
Obesity (N = 75)	16 (21.3%)	59 (78.7%)	0.368
First-line DMT (N = 268)	61 (22.8%)	207 (77.2%)	0.059
Copaxone (N = 28)	4 (14.3%)	24 (85.7%)	0.502
Dimethyl fumarate (N = 114)	23 (20.2%)	91(79.8%)	0.769
Interferon (N = 71)	23 (32.4%)	48 (67.6%)	0.003
Teriflunomide (N = 55)	11 (20%)	44 (80%)	0.873
Second-line DMT (N = 441)	75 (17%)	366 (83%)	0.059
Alemtuzumab (N = 69)	13 (18.8%)	56 (81.2%)	0.940
Cladribine (N = 43)	6 (14%)	37 (86%)	0.369
Fingolimod (N = 47)	8 (17%)	39 (83%)	0.687
Natalizumab (N = 124)	22 (17.7%)	102 (82.3%)	0.654
Ocrelizumab (N = 103)	19 (18.4%)	84 (81.6%)	0.838
Rituximab (N = 55)	7 (12.7%)	48 (87.3%)	0.206

PPMS: Primary progressive Multiple Sclerosis; RRMS: Relapsing remitting Multiple Sclerosis; SPMS: Secondary progressive Multiple Sclerosis.

## Data Availability

We have a database that was generated through a Redcap system with a link and a password for each of the participating centers. We are committed to safeguarding this data, and while it is available for external audits, these are proprietary data and cannot be accessed without our consent. For any inquiries regarding the data’s characteristics and accuracy, please contact the corresponding author.
